# The Effect of Dongeui Qigong for Prehypertension and Mild Essential Hypertension

**DOI:** 10.1155/2017/4274538

**Published:** 2017-10-19

**Authors:** Ji-Eun Park, Jung-Eun Kim, Soyoung Jung, Aeran Kim, Hyoju Park, Sanghoon Hong

**Affiliations:** ^1^Mibyeong Research Center, Korea Institute of Oriental Medicine, Daejeon, Republic of Korea; ^2^Clinical Research Division, Korea Institute of Oriental Medicine, Daejeon, Republic of Korea; ^3^Department of Internal Medicine, Dongeui Oriental Hospital of Dongeui University, Busan, Republic of Korea

## Abstract

Although several previous studies have reported the effect of qigong on lowering blood pressure, rigorous trials are lacking. Studies evaluating the effect of qigong on prehypertension are also scarce. This study aimed to assess the effect of qigong on prehypertension and mild hypertension. Participants with prehypertension or mild hypertension were randomized to the Dongeui qigong group or a nontreated control group. In the qigong group, Dongeui qigong was administered 5 times/week for 12 weeks. The control group did not receive any intervention for blood pressure control. Fifty-two participants were included in this study. Even though diastolic blood pressure was significantly reduced in the qigong group after 8 weeks (*p* = 0.04) compared to baseline, the difference in change in blood pressure between the qigong and control groups was not significant. There were no significant differences in quality of life between the qigong and control groups. Dongeui qigong is not significantly effective in pre/mild hypertension compared with controls. This result could be due to a lack of effect of qigong or caused by other factors, such as the type of qigong, target symptoms, inappropriate sample size, and compliance of participants.* Trial Registration*. This trial is registered with KCT0001397 (Clinical Research Information Service).

## 1. Introduction

Hypertension is a risk factor for cardiovascular disease, which is a leading cause of death worldwide [1]. In addition to patients with hypertension, those with a systolic blood pressure (SBP) of 120–139 mmHg or a diastolic blood pressure (DBP) of 80–89 mmHg (prehypertension) are at higher risk of hypertension and cardiovascular disease compared with those with normal blood pressure [2]. Therefore, prehypertension and hypertension require proper management of blood pressure.

To prevent hypertension, the World Health Organization recommends a healthy diet, quitting smoking, and more exercise [1]. However, lifestyle changes are not sufficient for some people, and achieving and maintaining these goals are difficult [3]. Additionally, medication for control of hypertension has some problems, such as side effects and cost of treatment [4].

For these reasons, many people use complementary and alternative medicine to control their blood pressure [5, 6]. Qigong is a complementary and alternative medicine and has been used for managing blood pressure [7]. Qigong prevents disease and improves health through flow of qi. A recent systematic review reported that qigong significantly reduced SBP and DBP compared with no intervention [7]. However, more rigorous, randomized, controlled trials are required. Another systematic review that assessed the effect of qigong for primary prevention of cardiovascular disease reported that qigong might reduce blood pressure, but the evidence was limited [8]. Moreover, many previous studies assessed the effect of qigong on hypertension but did not include prehypertension [7, 9]. Therefore, this study aimed to assess the effect of qigong on changes in blood pressure for prehypertension and mild hypertension using Dongeui qigong exercise.

## 2. Materials and Methods

### 2.1. Study Design and Ethics Approval

The study was a parallel, randomized, no treatment, controlled trial that was designed to compare qigong with no treatment for prehypertension and mild hypertension. This study was approved and conducted in the Oriental Medical Center of Daejeon University in the Republic of Korea. Full details of the trial protocol can be found at http://cris.nih.go.kr/.

### 2.2. Inclusion/Exclusion Criteria

Participants aged 19–65 years were included in the study. The participants had SBP between 120 and 159 mmHg and/or DBP between 80 and 99 mmHg. Participants were excluded if they had serious symptomatic cardiac disease, such as previous myocardial infarction, angina, or heart failure, or previous transient ischemic attacks or stroke. They were also excluded if they had secondary hypertension, were taking medications that could affect hypertension, such as stimulants or central nervous system depressants, and needed antihypertensive medications. Concomitant illness, including diabetes, cancer, infectious disease, renal failure, pregnancy or the possibility of pregnancy, moderate to high risk of cardiac complications during exercise, and inability to comprehend and complete the study assessments or to comply with the study instructions were also exclusion criteria.

Blood pressure was measured by a trained investigator. Blood pressure was measured three times with intervals of 5 minutes in the sitting position, and the average of three blood pressure measurements was used in analysis.

### 2.3. Recruitment and Randomization Procedures

The participants were recruited through advertisements in a local newspaper and bulletin board in the hospital. Randomization was performed by a statistician using a computerized randomization method. The participants were randomly assigned to either the qigong group or the control group with an assignment of 1 : 1 by clinical research coordinators. A sealed envelope was used to assign participants to groups. Blinding of patients and assessors was not possible because the control group had no intervention. However, the statistician was blinded to group allocation.

### 2.4. Intervention

Qigong used in this study was developed by experts on qigong who have more than 20 years of experience with qigong. The qigong focused on breathing and meditation to lower blood pressure and was termed Dongeui qigong. Each Dongeui qigong class lasted 50 minutes and consisted of a warm-up (15 minutes), main qigong treatment (25 minutes), and a cool-down (10 minutes) portion. Details of Dongeui qigong have been reported in a previous study by this research team [10]. However, in this study, the warm-up and cool-down exercise times were extended to focus on stabilization of autonomic nervous system activity.

When the participants were assigned to the qigong group, they attended a qigong class after randomization and were provided qigong by an investigator. After this time, they were given a video and pamphlet about qigong and asked to perform qigong treatment at home more than five times a week for 12 weeks. Every 2 weeks, they visited the hospital and took qigong with an investigator. They were also asked to check the number of qigong sessions that they completed in a qigong diary.

The control group did not receive any intervention for prehypertension or mild hypertension. Participants in the control group were also instructed to maintain their routine lifestyle during the study period.

### 2.5. Assessment

The primary outcome was changes in blood pressure before and after qigong exercises. The Measure Yourself Medical Outcome Profile (MYMOP2) and EQ5D were used to assess the quality of life. Additionally, body mass index (BMI) and the lipid profile were assessed.

The MYMOP2 questionnaire is a patient-generated or individualized outcome questionnaire, which is applicable to patients with physical, emotional, and social symptoms; [11] is problem-specific, but also includes general wellbeing.

EQ5D is a standardized measure of health status and it comprises five dimensions, including mobility, self-care, usual activities, pain/discomfort, and anxiety/depression. Each dimension has three levels (no problems, some problems, and extreme problems) [12]. Blood pressure and quality of life were assessed at baseline, after 4, 8, and 12 weeks, and at a 4-week follow-up. BMI was assessed at baseline and after 12 weeks.

### 2.6. Sample Size and Data Analysis

The sample size of each group was decided as 23 based on a pilot study conducted by this research team. In our pilot study, the dropout rate was 10%. Therefore, considering this dropout rate, the sample size needed to be 26 in each group, with a total of 52 participants.

The change in blood pressure at the end of the 12-week treatment period was the primary endpoint. The primary comparison was between the participants who were randomly assigned to the qigong group versus the control group.

All data were analyzed using the principle of intent-to-treat (ITT). Group differences in baseline characteristics were tested using the *χ*^2^ test for dichotomous variables and the *t*-test for continuous variables. For the continuous outcome measures, analysis of covariance was performed to compare the changes between the qigong and control groups. The paired *t*-test was used to compare the effects before and after treatment.

When the assumption of normality was violated, the Wilcoxon rank sum test and Wilcoxon signed rank test were used. Statistical analysis was performed using R software and the level of significance was established at *p* = 0.05.

## 3. Results

### 3.1. Demographic Data

A total of 74 participants were screened for eligibility, and 52 were included in this trial (four participants withdrew consent and another 18 participants did not meet the inclusion criteria). The eligible participants were randomly assigned to the qigong group (*n* = 25) or the control group (*n* = 27), and data for all these participants were analyzed.

**Figure 1:**
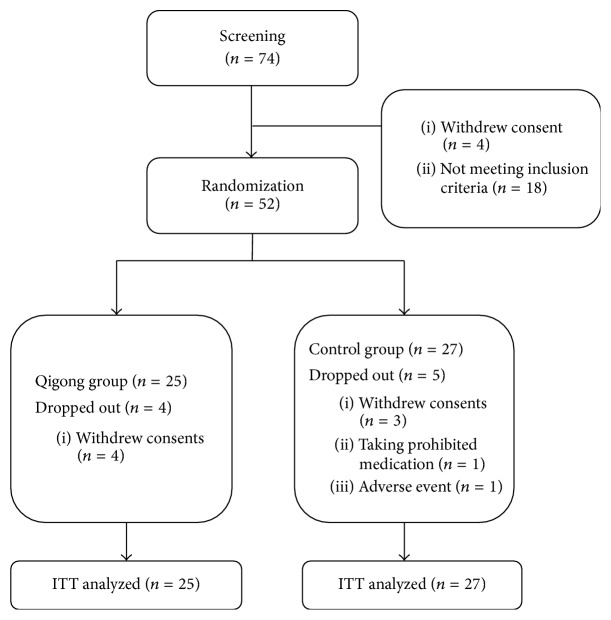
Flowchart of this study.

During study, four participants in the qigong group and three participants in the control group dropped out because they withdrew consent. One participant from the control group dropped out as a result of taking prohibited medication and another participant from the control group dropped out because of serious adverse event (hospitalization from acute cholecystitis). The subjects were recruited between September 16, 2014, and September 14, 2015, and the trial ended on January 8, 2016 (Figure 1).

Demographic data, including age and sex, were compared between the qigong and control groups. No significant differences in age, sex, temperature, the amount of smoking, and proportion of hypertension treatment were identified between participants in the two groups. Even though pulse was different between the two groups, it was not adjusted because the change in blood pressure might be not significantly affected. Blood pressure and the proportion of prehypertension participants were not significantly different between the qigong and control groups at baseline (Table 1).

### 3.2. Primary Efficacy Variable

The difference in blood pressure between the qigong and control groups was not significant at the end of qigong treatment or at the follow-up period. After 12 weeks of qigong treatment, the difference in change of blood pressure between the qigong and control groups was 3.58 mmHg (*p* = 0.31) for SBP and 1.73 mmHg (*p* = 0.57) for DBP. This difference was still not significant after a follow-up of 4 weeks (1.11 mmHg, *p* = 0.7 for SBP; 0.31 mmHg, *p* = 0.84 for DBP).

In the qigong group, SBP was marginally significantly decreased after 8 weeks (−5.0 mmHg, *p* = 0.057) and 12 weeks (−5.19 mmHg, *p* = 0.076). However, this reduction was not significant at the 4-week follow-up (*p* = 0.23). Control group also showed marginally significant reduction in SBP after 12 weeks (−5.61 mmHg, *p* = 0.094). The change of DBP was not significant in qigong or control group (Table 2).

### 3.3. Secondary Efficacy Variables

Total MYMOP2 scores were not significantly different between the qigong and control groups at 12 weeks (*p* = 0.19) or at the 4-week follow-up (*p* = 0.17). There was no significant difference in symptoms (*p* = 0.32), activity (*p* = 0.29), or wellbeing (*p* = 0.48) after 12 weeks between the qigong and control groups.

Among MYMOP2 categories, the total score and symptoms were significantly decreased in the qigong group after 8 weeks (total: *p* < 0.01, symptoms: *p* < 0.001), 12 weeks (total: *p* < 0.001, symptoms: *p* < 0.001), and the follow-up of 4 weeks (total: *p* = 0.01, symptoms: *p* < 0.001). In the control group, only symptoms were significantly decreased after 4 weeks (*p* = 0.04), 8 weeks (*p* < 0.01), and follow-up of 4 weeks (*p* = 0.02) (Table 3).

The qigong group did not show a significant change in EQ5D scores compared with the control group after 12 weeks (*p* = 0.31). EQ5D was not significantly different between baseline and 12 weeks in the qigong group (*p* = 0.16) or control group (*p* = 0.9) (Table 4). BMI was significantly different between the qigong and control groups after 12 weeks (*p* < 0.001). The reduction of BMI was significant in the control group (*p* = 0.04), but it was not significantly different in the qigong group (*p* = 0.1) (Table 5).

### 3.4. Safety

Seven participants (four in the qigong group and three in the control group) withdrew their consent and dropped out, and one participant in the control group dropped out because he took prohibited medication. There were no reported adverse events in this trial. One participant in the control group reported hospitalization during the trial, but it was due to acute cholecystitis and did not appear to be related to qigong.

## 4. Discussion

A previous systematic review showed an effect of qigong on hypertension [7]. However, another recent review suggested limited evidence of this effect because previous studies that assessed the effect of qigong had a high risk of bias and required more rigorous studies [8]. Our study also showed no significant effect of qigong on prehypertension and mild hypertension compared with the no-intervention control group. This insignificant result could have been caused by factors of qigong (real effect, type of qigong), study design (inclusion criteria), or participants (compliance).

Qigong does not appear to be effective for lowering blood pressure. Several previous studies have shown that qigong is effective in decreasing blood pressure compared with no treatment [7, 13, 14]. However, many studies on qigong were still of low quality and had bias [9]. The current study was more rigorous than previous studies that were not randomized [15], without allocation concealment [16, 17] or with no control group [18]. The results of our randomized, controlled, statistician-blinded study might be due to the real effect of qigong. Further studies with rigorous methodology are required to assess the effect of qigong on lowering blood pressure.

Our negative results could have been caused by the type or duration of qigong. The effects of qigong could be different by the type of qigong. Lee et al. assessed the effect of internal and external qigong for controlling pain. They reported that the effect of internal qigong was not convincing [19], although external qigong was significantly effective [20]. In previous studies, Tai chi, which is Chinese conditioning exercise focusing on graceful movement like qigong [21], and Baduanjin exercise [22] also showed encouraging evidence for hypertension. The type of qigong used in previous studies for hypertension varied from seated qigong [18], Mawangdui Daoyinshu [23], Baduanjin [24], and qigong exercise [25]. Dongeui qigong used in this study was developed by consensus of qigong experts and literature review, but it could cause insignificant result if it is not suitable for hypertension.

Additionally, the duration of qigong in this study, which was 12 weeks, was shorter than that in many previous studies [7]. A recent systematic review showed that qigong treatment ranged from 8 weeks to 12 months, and 10 of 20 included studies reported a duration of qigong treatment longer than 3 months [7]. Therefore, further study of the effect of qigong on hypertension is recommended, using a qigong program longer than 3 months, and the qigong program needs to be described in detail.

Even though qigong is effective for hypertension, this effect was not significant in prehypertension and mild hypertension in our study. Most previous studies that showed an effect of qigong on lower blood pressure included patients with hypertension [7, 9, 13]. Only a few previous studies investigated the effect of qigong on prehypertension or mild hypertension. One study reported a significant effect of qigong on prehypertension and mild hypertension [25]. Further studies targeting prehypertension and mild hypertension are required because control of blood pressure is required in these conditions.

Compliance of participants could affect qigong treatment. In our study, participants were asked to take qigong five times a week and fill in a diary to describe this treatment. If participants had qigong treatment less than 42 times during the study, they dropped out. A total of 15 of 25 participants in the qigong group (60%) reported that they conducted qigong treatment five times or more per week, even though they had this treatment more than 42 times for 12 weeks. Our compliance rate was low compared with clinical trials that used medication with a rate of 83–94% [26], as well as a previous study on qigong that showed adherence of 79% of participants [27]. Another previous study on qigong for breast cancer also reported a compliance of 89.6% in participants who had group qigong sessions and 78.5% for home qigong exercise [28]. This low compliance could be the reason for an insignificant effect of qigong. Considering the possibility of overestimation of self-reported compliance [29] or a discrepancy between self-reported and verified compliance [30], the proportion of participants actually satisfying the qigong regimen might be less than 60% [30].

When we analyzed the association between compliance and change in blood pressure, we found that the change in blood pressure was higher in the good compliance group (SBP: −8.46 mmHg, DBP: −3.03 mmHg) compared to the bad compliance group (SBP: 1.89 mmHg, DBP: 3.11 mmHg), even though the difference was not significant. In addition, blood pressure was significantly lower after 12 weeks compared to baseline in the good compliance group (SBP: *p* = 0.019, DBP: *p* = 0.059), but not in the bad compliance group (SBP: *p* = 0.7, DBP: *p* = 0.46). Hence, participants' compliance is very important in a qigong trial, and researchers should focus on developing methods to achieve good participant compliance.

Many systematic reviews have reported that the quality of qigong studies is low and the evidence on the effect of qigong is limited [8]. Further studies are required to assess the effect of qigong considering the limitations of this study as follows. First, further studies should decide on the type and frequency of qigong treatment based on sufficient evidence, including experts' consensus and a literature review. Second, more studies on qigong need to be conducted, including participants with prehypertension and mild hypertension and a comparison of the effect of qigong between mild hypertension and hypertension. Third, an accurate sample size needs to be decided based on previous studies on qigong. Fourth, compliance of qigong should be assessed using a more objective and valid tool rather than self-reporting, and various methods need to be developed to encourage participants' compliance.

## 5. Conclusion

Dongeui qigong treatment for 12 weeks is not significantly effective in prehypertension and mild hypertension compared with no treatment. Further randomized, clinical trials with a large sample size on prehypertension and mild hypertension should be conducted.

## Figures and Tables

**Table 1:** Demographic data of participants in each group.

	Qigong group (*n* = 25)	Control group (*n* = 27)	*p* value
Age (years)	54.52 ± 6.96	52.93 ± 8.45	0.46
Sex^§^ (males/females)	13/12	22/5	0.05
Temperature	36.76 ± 0.25	36.71 ± 0.24	0.46
Pulse	65.88 ± 6.04	70.23 ± 8.49	0.04
Amount of smoking	15.00 ± 8.66	10.33 ± 8.02	0.48
Treatment of hypertension			
Yes	1	3	0.61
No	24	24	—
Blood pressure			
Systolic	134.45 ± 10.41	130.47 ± 13.93	0.25
Diastolic	85.23 ± 6.43	85.41 ± 7.83	0.93
Hypertension stage			
Prehypertension	15	15	0.97
Mild hypertension	10	12	

^§^Chi-square test. The other measures were analyzed using analysis of covariance adjusted for group and center.

**Table 2:** Changes in blood pressure in each group.

	Qigong group	Change of BP in qigong group	Control group	Change of BP in control group
Baseline				
SBP	134.45 ± 10.41		130.47 ± 13.93	
DBP	85.23 ± 6.43		85.41 ± 7.83	
After 4 weeks				
SBP	132.67 ± 11.61	−2.48 ± 11.25	132.24 ± 10.38	0.81 ± 10.08
DBP	84.62 ± 6.35	−1.53 ± 4.83	87.36 ± 7.29	2.09 ± 8.04
After 8 weeks				
SBP	129.63 ± 10.14	−5.0 ± 10.42	128.58 ± 10.92	−3.36 ± 12.88
DBP	84.15 ± 6.96	−1.81 ± 5.53	83.80 ± 5.53	−2.06 ± 7.78
After 12 weeks				
SBP	129.82 ± 9.11	−5.19 ± 12.06	126.48 ± 12.0	−5.61 ± 14.97
DBP	85.16 ± 7.60	−1.09 ± 7.18	83.92 ± 7.67	−1.83 ± 8.02
After f/u 4 weeks				
SBP	131.19 ± 12.52	−3.82 ± 13.34	129.68 ± 14.01	−2.57 ± 13.85
DBP	84.70 ± 9.31	−1.54 ± 8.02	84.19 ± 7.90	−1.37 ± 8.82

SBP: systolic blood pressure; DBP: diastolic blood pressure.

**Table 3:** Changes in MYMOP2 score in each group.

	Qigong group	Change of MYMOP2 score in qigong group	Control group	Change of MYMOP2 score in control group
Baseline				
Total	2.32 ± 1.07	—	2.22 ± 1.11	—
Symptoms	2.75 ± 1.07	—	3.00 ± 1.15	—
Activity	3.67 ± 1.37	—	1.33 ± 0.58	—
Wellbeing	2.00 ± 1.32	—	1.88 ± 1.39	—
4 wks				
Total	1.95 ± 0.75	−0.25 ± 0.78	2.17 ± 1.13	−0.17 ± 1.10
Symptoms	2.17 ± 1.04	−0.44 ± 1.04	2.50 ± 1.34	−0.57^*∗*^ ± 0.94
Activity	1.80 ± 1.30	−1.0 ± 0.82	1.67 ± 1.15	0.33 ± 1.53
Wellbeing	1.85 ± 0.99	−0.15 ± 1.31	2.27 ± 1.35	0.32 ± 1.40
8 wks				
Total	1.65 ± 0.56	−0.43^*∗*^ ± 0.65	1.85 ± 1.08	−0.49 ± 1.32
Symptoms	1.57 ± 0.76	−1.07^*∗∗∗*^ ± 0.92	1.77 ± 1.09	−1.38^*∗∗*^ ± 1.33
Activity	1.50 ± 0.71	−1.5 ± 2.12	1.50 ± 2.12	0.5 ± 2.12
Wellbeing	1.61 ± 0.61	−0.17 ± 1.15	1.95 ± 1.25	−0.15 ± 1.42
12 wks				
Total	1.62 ± 0.96	−0.68^*∗∗∗*^ ± 0.67	2.06 ± 1.23	−0.34 ± 1.47
Symptoms	1.67 ± 1.11	−1.13^*∗∗∗*^ ± 0.83	2.17 ± 1.64	−1.0 ± 1.71
Activity	2.33 ± 1.53	−1.33 ± 1.53	0.50 ± 0.71	−0.5 ± 0.71
Wellbeing	1.72 ± 1.13	−0.28 ± 0.96	2.00 ± 1.14	−0.2 ± 1.51
f/u 4 wks				
Total	1.63 ± 0.72	−0.60^*∗∗*^ ± 0.85	2.05 ± 1.15	−0.27 ± 1.54
Symptoms	1.73 ± 1.22	−1.07^*∗∗∗*^ ± 0.96	1.92 ± 1.31	−1.25^*∗*^ ± 1.60
Activity	2.33 ± 1.15	−1.33 ± 2.08	2.00 ± 1.41	1.0 ± 1.41
Wellbeing	1.58 ± 0.51	−0.37 ± 1.30	2.05 ± 1.12	−0.05 ± 1.54

^*∗*^ *p* < 0.05, ^*∗∗*^*p* < 0.01, and ^*∗∗∗*^*p* < 0.001.

**Table 4:** Changes in EQ5D in each group.

	Qigong group	Change of EQ5D score in qigong group	Control group	Change of EQ5D score in control group
Baseline	0.92 ± 0.04		0.93 ± 0.05	
Change of 4 weeks	0.93 ± 0.03	0.01 ± 0.04	0.92 ± 0.04	−0.01 ± 0.06
Change of 8 weeks	0.95 ± 0.02	0.02 ± 0.05	0.92 ± 0.05	−0.01 ± 0.06
Change of 12 weeks	0.94 ± 0.03	0.01 ± 0.03	0.93 ± 0.04	−0.002 ± 0.06
Chang of f/u 4 weeks	0.93 ± 0.04	0.01 ± 0.03	0.92 ± 0.06	−0.01 ± 0.06

**Table 5:** Changes in BMI in each group.

	Qigong group	Change of BMI in qigong group	Control group	Change of BMI in control group
Baseline	25.79 ± 3.18		24.50 ± 3.2	
Change of 12 weeks	25.50 ± 3.39	–0.19 ± 0.42	24.15 ± 3.24	–0.20^*∗*^ ± 0.44

^*∗*^ *p* < 0.05.

## References

[1] WHO, “A global brief on hypertension”, 2013

[2] Ishikawa Y., Ishikawa J., Ishikawa S., Kario K., Kajii E. (2017). Progression from prehypertension to hypertension and risk of cardiovascular disease. *Journal of Epidemiology*.

[3] Faries M. D., Abreu A. (2017). Medication Adherence, When Lifestyle Is the Medicine. *American Journal of Lifestyle Medicine*.

[4] Johnston A., Stafylas P., Stergiou G. S. (2010). Effectiveness, safety and cost of drug substitution in hypertension. *British Journal of Clinical Pharmacology*.

[5] Nahas R. (2008). Complementary and alternative medicine approaches to blood pressure reduction: an evidence-based review. *Canadian Family Physician*.

[6] Saydah S. H., Eberhardt M. S. (2006). Use of complementary and alternative medicine among adults with chronic diseases: United States 2002. *The Journal of Alternative and Complementary Medicine*.

[7] Xiong X., Wang P., Li X., Zhang Y. (2015). Qigong for hypertension: a systematic review. *Medicine*.

[8] Hartley L., Lee M. S. O., Kwong J. S. W. (2015). Qigong for the primary prevention of cardiovascular disease. *Cochrane Database of Systematic Reviews*.

[9] Lee M. S., Pittler M. H., Guo R., Ernst E. (2007). Qigong for hypertension: A systematic review of randomized clinical trials. *Journal of Hypertension*.

[10] Park J.-E., Liu Y., Park T. (2011). A trial for the use of qigong in the treatment of pre and mild essential hypertension: A study protocol for a randomized controlled trial. *Trials*.

[11] MYMOP *MYMOP*. http://www.bris.ac.uk/primaryhealthcare/resources/mymop.

[12] Foundatin E. R. EQ-5D. http://www.euroqol.org/about-eq-5d/how-to-use-eq-5d.html.

[13] Guo X., Zhou B., Nishimura T., Teramukai S., Fukushima M. (2008). Clinical effect of Qigong practice on essential hypertension: a meta-analysis of randomized controlled trials. *The Journal of Alternative and Complementary Medicine*.

[14] Kuang A. K., Wang C. X., Zhao G. S. (1986). Long-term observation on qigong in prevention of stroke--follow-up of 244 hypertensive patients for 18-22 years. *Journal of Traditional Chinese Medicine*.

[15] Lee M.-S., Lim H.-J., Lee M. S. (2004). Impact of qigong exercise on self-efficacy and other cognitive perceptual variables in patients with essential hypertension. *The Journal of Alternative and Complementary Medicine*.

[16] Ritter C., Aldridge D. (2001). Qigong Yangsheng as a therapeutic approach for the treatment of essential hypertension in comparison with a western muscle relaxation therapy: A randomised, controlled pilot study. *Chinesische Medizin*.

[17] Li W., Xing Z., Pi D. (1997). Influence of qi-gong on plasma TXB2 and 6-keto-PGF1 alpha in two TCM types of essential hypertension. *Hunan Yi Ke Da Xue Xue Bao*.

[18] Freeman S. R., Hanik S.-A. E., Littlejohn M. L. (2014). Sit, breathe, smile: Effects of single and weekly seated Qigong on blood pressure and quality of life in long-term care. *Complementary Therapies in Clinical Practice*.

[19] Lee M. S., Pittler M. H., Ernst E. (2009). Internal qigong for pain conditions: a systematic review. *The Journal of Pain*.

[20] Lee M. S., Pittler M. H., Ernst E. (2007). External Qigong for Pain Conditions: A Systematic Review of Randomized Clinical Trials. *The Journal of Pain*.

[21] Wang J., Feng B., Yang X. (2013). Tai Chi for essential hypertension. *Evidence-Based Complementary and Alternative Medicine*.

[22] Xiong X., Wang P., Li S., Zhang Y., Li X. (2015). Effect of Baduanjin exercise for hypertension: a systematic review and meta-analysis of randomized controlled trials. *Maturitas*.

[23] Chen D. (2016). Effect of Health Qigong Mawangdui Daoyinshu on Blood Pressure of Individuals with Essential Hypertension. *Journal of the American Geriatrics Society*.

[24] Xiao C., Yang Y., Zhuang Y. (2016). Effect of health Qigong Ba Duan Jin on blood pressure of individuals with essential hypertension. *Journal of the American Geriatrics Society*.

[25] Park J. E., Hong S., Lee M. (2014). Randomized, controlled trial of qigong for treatment of prehypertension and mild essential hypertension. *Alternative Therapies in Health and Medicine*.

[26] Sugimoto D., Myer G. D., Bush H. M., Hewett T. E. (2014). Effects of compliance on trunk and hip integrative neuromuscular training on hip abductor strength in female athletes. *The Journal of Strength and Conditioning Research*.

[27] Martínez N., Martorell C., Espinosa L., Marasigan V., Domènech S., Inzitari M. (2015). Impact of Qigong on quality of life, pain and depressive symptoms in older adults admitted to an intermediate care rehabilitation unit: a randomized controlled trial. *Aging Clinical and Experimental Research*.

[28] Liu W., Schaffer L., Herrs N., Chollet C., Taylor S. (2015). Improved sleep after Qigong exercise in breast cancer survivors: A pilot study. *Asia-Pacific Journal of Oncology Nursing*.

[29] Dieltjens M., Braem M. J., Vroegop A. V. M. T. (2013). Objectively measured vs self-reported compliance during oral appliance therapy for sleep-disordered breathing. *CHEST*.

[30] Kaunitz A. M., Portman D., Westhoff C. L., Archer D. F., Mishell D. R., Foegh M. (2015). Self-reported and verified compliance in a phase 3 clinical trial of a novel low-dose contraceptive patch and pill. *Contraception*.

